# A Case Series of Right Coronary Artery Anomalies With a Malignant Course

**DOI:** 10.7759/cureus.90534

**Published:** 2025-08-19

**Authors:** Malek Othman, Maimona Weisy, Montaser Al Smady, Ahmed Hasan, Yusuf Hallak, Hamza Alkowatli, Omar A Habboub, Ahmad Hallak

**Affiliations:** 1 Internal Medicine, Bridgeport Hospital, Bridgeport, USA; 2 Medicine and Surgery, Istinye University, Istanbul, TUR; 3 Internal Medicine, MedStar Health Georgetown University, Baltimore, USA; 4 Internal Medicine, Tawam Hospital, Abu Dhabi, ARE; 5 Internal Medicine, University of Toledo, Toledo, USA; 6 Internal Medicine, Hospital Corporation of America (HCA) Florida Blake Hospital, Bradenton, USA; 7 Cardiology, Ochsner Medical Center, New Orleans, USA

**Keywords:** anomalous coronary artery origin, case series, malignant coronary artery course, nstemi, right coronary artery, stemi

## Abstract

Anomalous aortic origin of the right coronary artery (AAORCA) is a rare congenital anomaly. Although most patients with this anomaly remain asymptomatic, it is well established that this anomaly may lead to angina, myocardial infarction (MI), or sudden cardiac death (SCD) in the absence of atherosclerotic disease. In this article, we report three cases in the Middle East that presented with syncope in two patients, as well as diaphoresis and ECG changes during a nasal septoplasty surgery in the third. All patients were diagnosed with AAORCA with interarterial course using coronary computed tomography angiography (CTA) after initial evaluation. Although medical management varied over the three cases, all patients tolerated medical management and were referred for surgical interventions. One patient underwent a surgical procedure, with positive outcome afterwards. It is important to recognize the atypical presentations of the malignant course of AAORCA and demonstrate the importance of medical and surgical management in this disease, specially in understudied areas such as the Middle East.

## Introduction

Coronary artery anomalies (CAAs) are a group of developmental abnormalities that describe an aberrant origin, course or termination that can occur in epicardial coronary arteries [1]. CCA remain the second most common cause of sudden cardiac death (SCD) in young athletes [2]. Although relatively rare, anomalous aortic origin of a coronary artery (AAOCA) is the most common type of CAA in which coronary arteries follow an unusual origin pattern from the aorta [3,4]. AAOCA includes several subtypes, including anomalous aortic origin of the right and left coronary arteries (AAORCA and AAOLCA) [5]. The presentation of an anomalous right coronary artery (ARCA) is more commonly seen than an anomalous left coronary artery (ALCA) [6]. In AAORCA, the right coronary artery originates from the left coronary sinus and can follow several courses [5]. The malignant course of AAORCA represents the interarterial path where the RCA passes between the aorta and the pulmonary artery, in which compression between major vessels can lead to ischemia in the tissues that are supplied by the right coronary artery [7]. This can manifest in myocardial infarction (MI), ventricular arrhythmias, and SCD. In this case series, we report three patients diagnosed with AAORCA with a malignant course, highlighting the importance of prompt identification and appropriate treatment in similar clinical scenarios. This case series will help in raising the awareness of atypical presentations of AAORCA, especially in the Middle East, and the importance of adherence to current recommendations regarding surgical and conservative approaches in symptomatic cases.

## Case presentation

Patient 1

**Figure 1:**
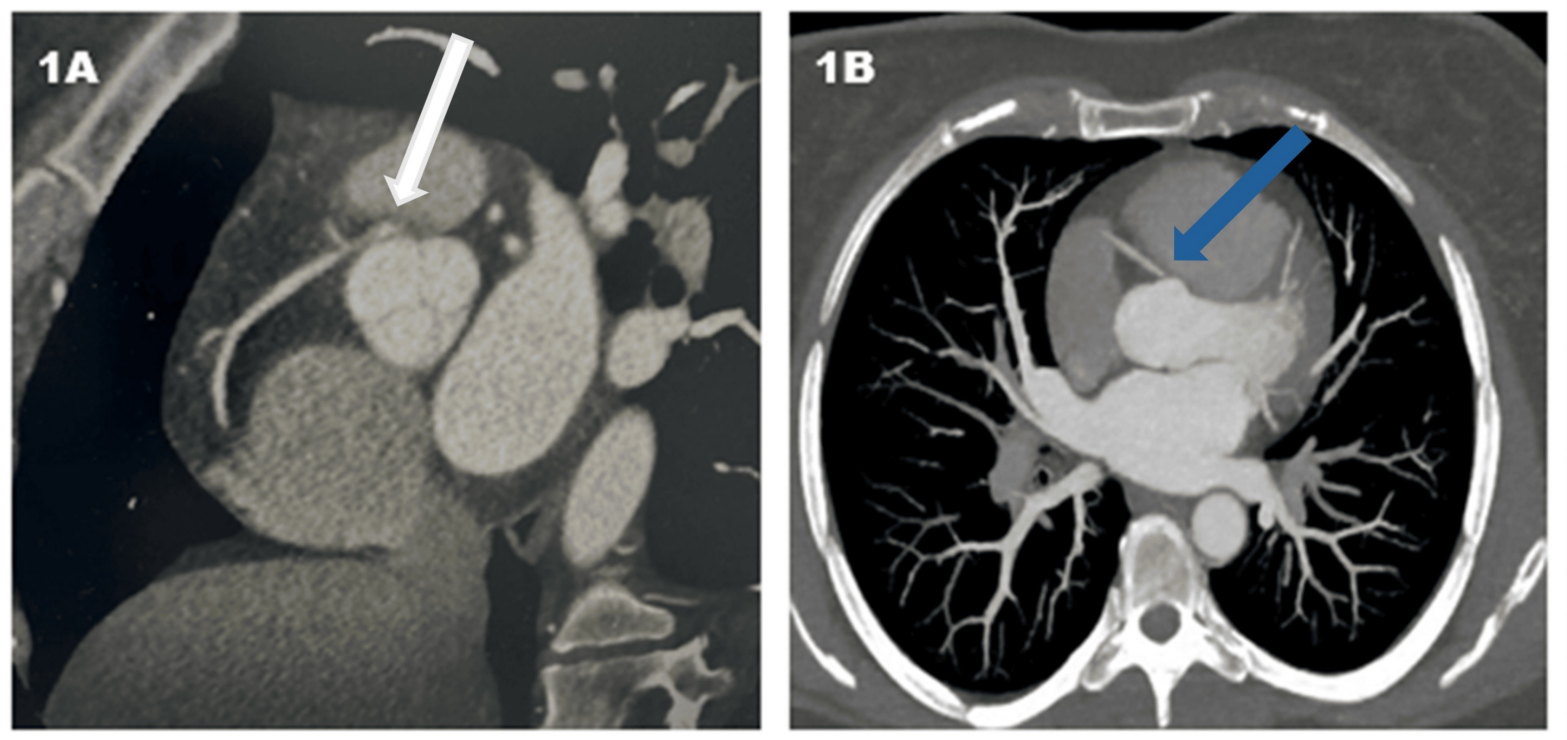
Case 1 Imaging (A) Coronary computed tomography angiography (CTA) showing right coronary artery originating from the left coronary cusp with an anterior course with compression between the aorta and right ventricular outflow tract (white arrow). (B) Chest CTA showing anterior course of right coronary artery (RCA) originating from the left coronary cusp (blue arrow).

A 40-year-old female patient presented after a syncopal episode upon standing up to get out of bed with preceding retrosternal burning pain and diaphoresis, followed by lightheadedness. Vital signs were within normal limits upon presentation and physical examination was unremarkable. Troponin I was elevated at 0.28 ng/ml. Electrocardiogram (ECG) showed normal sinus rhythm with no ischemic changes. The patient underwent coronary angiography (CAG), which showed no obstructive coronary lesions but noted anterior/left-sided originating RCA. Coronary computed tomography angiography (CCTA) confirmed AAORCA originating from the left coronary cusp and taking an anterior course between the aorta and pulmonary artery (Figure 1A, 1B). The patient was started on Metoprolol, was informed to restrict exercise and referred for surgery in another institute. Upon two-month follow-up, the patient was symptom-free, but surgery was pending.

Patient 2

**Figure 2:**
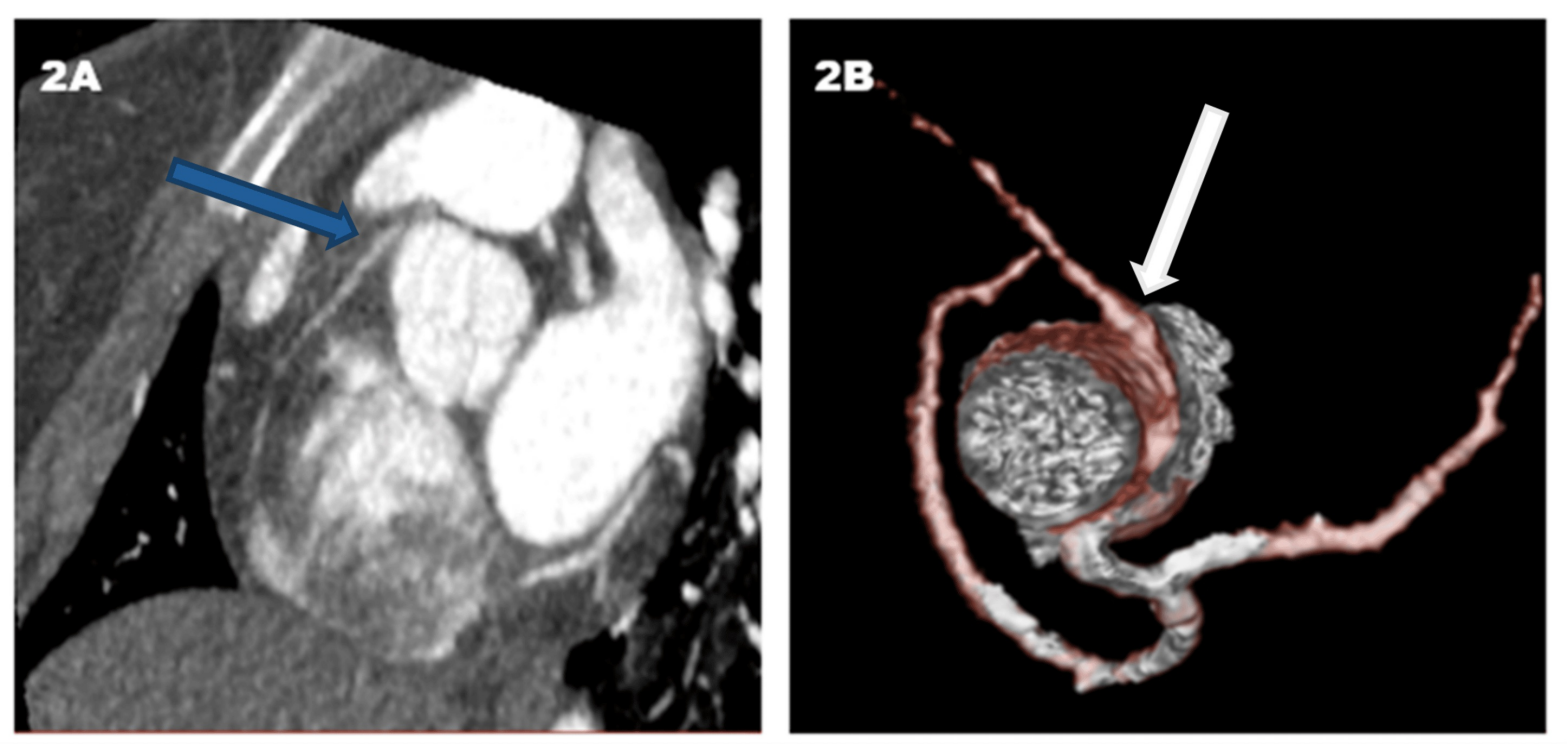
Case 2 Imaging (A) Cardiac computed tomography angiography (CTA) cross-view showing right coronary artery (RCA) coursing between the aorta and pulmonary trunk (blue arrow). (B) Three-dimensional reconstruction image showing the smaller diameter RCA when compared with the left coronary artery (LCA) due to compression of the RCA between the aorta and pulmonary trunk (white arrow). Image credit for B: Dr. Malek Othman.

A 17-year-old female patient presented after a syncopal episode while taking a shower after exercising with preceding chest pain, but no palpitations, light-headedness, nausea, or diaphoresis. Vital signs were within normal limit and physical examination was unremarkable. Troponin T, creatinine kinase-MB (CK-MB), and other lab work were within normal limits. ECG showed ST-elevations in the inferior leads. Transesophageal echocardiography (TEE) showed minimal hypokinesis of the inferior wall with normal ejection fraction. The patient refused cardiac catheterization. She was treated for myocarditis with non-steroidal anti-inflammatory drugs (NSAIDs). Her chest pain improved slightly but persisted. Further evaluation with cardiac magnetic resonance imaging (CMRI) showed evidence of an inferior wall infarct. CCTA confirmed AAORCA originating from the left coronary cusp with the course passing intramurally between the great vessels (Figure 2A, 2B). Subsequently, she underwent surgery with unroofing of the RCA and moving of the bifurcation of the pulmonary artery towards the left pulmonary artery. The patient tolerated surgery well and had an uncomplicated hospital course. The patient remained asymptomatic at a six-month follow-up.

Patient 3

**Figure 3:**
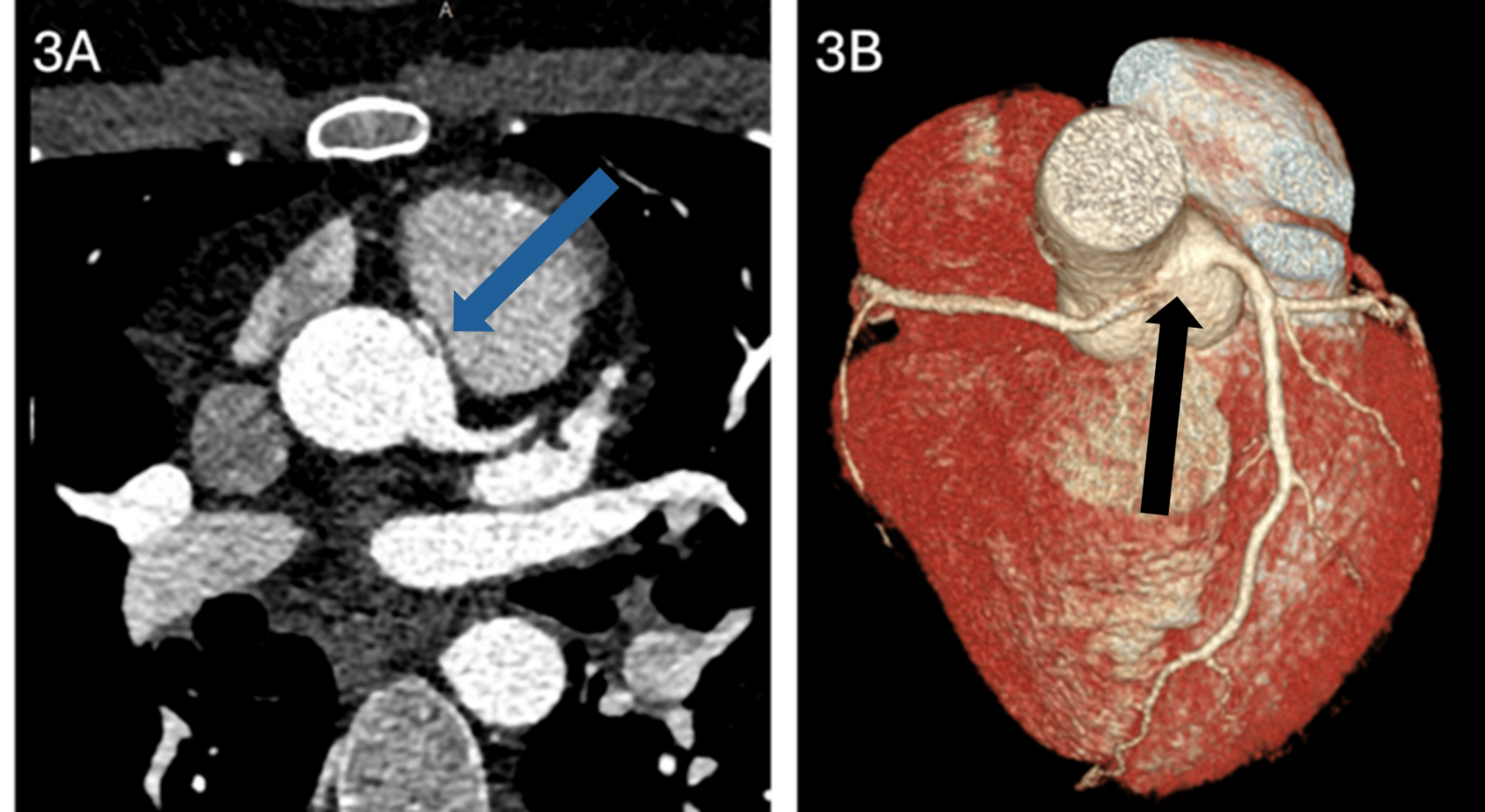
Case 3 Imaging (A) Cardiac computed tomography angiography (CTA) cross-view showing right coronary artery (RCA) originating from the left aortic sinus (blue arrow). (B) Three-dimensional reconstruction image showing the RCA course anterior to the ascending aorta (black arrow). Image credit for B: Dr. Malek Othman.

A previously healthy 30-year-old male patient underwent septoplasty under general anesthesia in a secondary-level hospital during which he started bleeding profusely from the operation site. Local adrenaline injection into the operation site was used to control the bleeding. Afterwards, he developed tachycardia and ECG showed ST depression in several leads and T wave inversion in leads V1-V2. Troponin T was 0.55 ng/ml. He was referred to a tertiary-level hospital for further evaluation and management. Upon admission, the patient had no chest pain or shortness of breath but was sweating profusely. On examination, the patient was tachycardic but otherwise vitally stable. Chest and cardiovascular examinations were unremarkable. Laboratory investigations, including hemoglobin level, platelet count, and international normalized ratio (INR) were within normal limits. Troponin T was 0.51 ng/ml. Transthoracic echocardiogram (TTE) was unremarkable with no regional wall motion abnormality. He was treated as a case of non-ST-elevation myocardial infarction (NSTEMI) with dual antiplatelets and atorvastatin. Coronary angiography (CAG) showed an abnormal origin of the RCA with no significant atherosclerotic disease. CCTA confirmed AAORCA, originating from the left coronary cusp with slit-like origin and interarterial course (Figure 3A, 3B). Sweating stopped and he remained pain-free during the hospital course. Troponin decreased gradually to 0.11 ng/ml over two days. He was discharged on dual antiplatelet and atorvastatin with scheduled outpatient stress nuclear test in four weeks and was referred for surgical intervention at another institute. On one-month follow-up, the patient remained asymptomatic.

## Discussion

Coronary artery anomalies can be defined by an abnormal origin or course of the RCA, left anterior descending artery or the left circumflex coronary artery, and have been seen in less than 1% of the general population [4]. Anomalous aortic origin of a coronary artery is rare but is the most common CAA [3]. Subtypes of AAOCA include AAORCA and AAOLCA, with a prevalence in the general population of 0.06%-0.9% and 0.02%-0.1%, respectively [6].

The RCA arises from the right sinus of Valsalva of the ascending aorta, continuing anteriorly and to the right, between the auricles and pulmonary arteries, descending in the right atrioventricular sulcus [8]. In an AAORCA, the right coronary artery originates from the left coronary sinus and can follow several courses: interarterial, prepulmonic, subpulmonic, retroaortic, and retrocardiac [5]. In an interarterial manifestation, the right coronary artery courses between the great vessels, the aorta and pulmonary trunk, and is associated with MI, ventricular arrhythmias, and SCD, in the absence of coronary artery diseases (CADs) [4]. The incidence of AAORCA with an interarterial course varies between 0.026% and 0.25% [9]. On exercise nuclear testing, AAORCA with interarterial course was shown to cause reversible defects in the RCA tissue, which is consistent with reversible ischemia [10].

Many pathophysiologic mechanisms of this association have been suggested. The mechanical compression of the right coronary artery by the great vessels is the usual explanation [4]. During exercise, in addition to the myocardial tissue demand, there is an increase in stroke volume, increasing the diameter of the great vessels, leading to compression of the right coronary artery. Furthermore, the oblique angle at the segregation of the RCA and the left coronary sinus produces a slit-like orifice in the aortic wall, which could collapse during exertion [4]. Lastly, it is suggested that the proximal portion of the anomalous RCA is prone to spasm more than normal coronary vessels [4].

In our series, all three cases exhibited symptoms following an increase in myocardial oxygen demand, showing the functional significance of AAORCA. In the first case, sudden standing likely activated the baroreflex, which triggers the sympathetic nervous system, therefore leading to compression of the anomalous vessel. The second case developed symptoms post-exercise. In the third case, intraoperative epinephrine injection simulated exertional stress. Despite the different instances, all cases shared a common pathway, an increased demand along with compromised coronary perfusion due to interarterial compression. These findings support the proposed pathophysiologic mechanisms cited in the literature.

Diagnostics

Several diagnostic imaging techniques may be used to assess coronary anatomy and the presence of high-risk features. These include echocardiography, coronary computed tomography angiography (CCTA), cardiovascular magnetic resonance (CMR), and coronary angiography (CAG). CCTA has been established as the current gold standard technique in the assessment of CAA [1]. It provides a detailed characterization of the anatomic evidence associated with high-risk CAAs and allows the visualization of the cardiac and non-cardiac surrounding structures, as well as their relative three-dimensional relationship [11,12].

CMR provides a similar anatomic definition of the origin and course of coronary arteries to that seen in CCTA while also evaluating both systolic and valvular function and characterization of the myocardial tissue [13]. Gadolinium enhancement may also reveal the presence of fibrosis, which may establish an association between the anomaly and myocardial ischemia [14]. This highlights the emerging role of CMR as an alternative to CCTA, yet due to lower spatial resolution and lower availability of expertise and specific instrumentation [15]. It has been attributed to have a secondary role in the investigation of CAAs in contrast to the main role of CCTA [16].

Echocardiography plays a minor role in adults with CAAs due to the lower diagnostic accuracy in identifying coronary ostia and the inability to visualize the course of the great vessels [13]. However, it may be the initial investigation providing an incidental finding, which is further investigated by other modalities.

Provocative stress testing, such as exercise or pharmacological stress imaging with dobutamine or vasodilators, may be used to assess inducible ischemia. However, the incident or life-threatening events in patients with CAAs have been associated with strenuous effort [17]. Furthermore, the sensitivity of stress testing to identify patients at risk of myocardial ischemia secondary to CAAs is low [6].

Management and prognosis

There is a consensus that patients experiencing AAOCA-related symptoms should undergo activity restriction and surgical intervention. These symptoms include instances of syncope associated with ventricular arrhythmia, chest pain (consistent with angina), high-risk ambient ventricular arrhythmias, SCD, and evidence of ischemia on provocative testing [18].

There are different opinions on the management of AAOCAs. AAOLCA diagnosis prompts surgical intervention, with/without symptoms, while surgery is only indicated for AAORCA diagnosis when symptoms are present [19]. These interventions include medical and conservative treatments such as sport restriction, surgery, and percutaneous coronary intervention (PCI), which is considered a safe and successful alternative, but with limited long-term follow-up data [20]. Of our patients, one patient underwent a surgical correction while the other two receievd medical therapy, while awaiting surgery.

One of the more common surgical approaches is the unroofing technique, which is performed when the intramural segment can be identified [19]. When the intramural course cannot be identified, pulmonary artery translocation is performed. This technique is reserved for patients with an anomalous right coronary originating from a single coronary ostium and an anterior translocation [19] .

Long-term follow-up with patients that have undergone surgery for AAOCA is important to understand the associated risks, but in published series, the risk is extremely low, with excellent intermediate-term survival [18]. Medical therapy is deemed to be safe, despite lack of evidence supporting the use of beta-blockers18. In our first case, we used beta-blockers and only allowed the patient to do light, non-strenuous activities.

## Conclusions

This case series highlights the variable presentations and critical importance of timely diagnosis in patients with AAORCA with interarterial course. The cases underscore the necessity of an individualized approach to management, including medical therapy, surgical intervention, and adherence to exercise restrictions. These managements mitigate the risk of adverse outcomes such as myocardial infarction, ventricular arrhythmias and sudden cardiac death. Increasing awareness and understanding of AAORCA can enhance clinical outcomes through prompt recognition and appropriate treatment strategies.
